# Antimicrobial Activity of Selected Red and White Wines against *Escherichia coli*: In Vitro Inhibition Using Fish as Food Matrix

**DOI:** 10.3390/foods9070936

**Published:** 2020-07-15

**Authors:** Heidi Christine Santoro, Danijela Skroza, Anđela Dugandžić, Mladen Boban, Vida Šimat

**Affiliations:** 1 Department of Pharmacology, School of Medicine, University of Split, Šoltanska 2, HR-21000 Split, Croatia; heidi.christine.santoro@gmail.com (H.C.S.); mladen.boban@mefst.hr (M.B.); 2 Department of Food Technology and Biotechnology, Faculty of Chemistry and Technology, University of Split, Ruđera Boškovića 35, HR-21000 Split, Croatia; angy.duga96@gmail.com; 3 Department of Marine Studies, University of Split, Ruđera Boškovića 37, HR-21000 Split, Croatia

**Keywords:** wine, sea bass, antimicrobial activity, MIC, phenols

## Abstract

Five different wines (standard Graševina, macerated Graševina with and without sulfur, rosé, and standard Plavac Mali), all typical Croatian wines, were tested to determine the antimicrobial activity against two *Escherichia coli* bacterial strains (ATCC^®^ 25922 and ATCC^®^ 8739) in vitro and using sea bass (*Dicentrarchus labrax*) fillets as food matrix. The chemical composition of wines (pH, acidity, alcohol, total phenolics, anthocyanins, tannins, and sulfur content) and antimicrobial activity (minimal inhibitory concentration (MIC), agar-well diffusion method) were determined. The total phenolic content of the wines ranged from 305–3210 mg gallic acid equivalents per liter (GAE/L), and did not correlate to antimicrobial activity. The two wines with the lowest phenolic content (standard Graševina and rosé) had the lowest MIC values (122 and 429 mg GAE/L). A specific relation between the winemaking process and a particular MIC value was not established. There was also no relation found between the pH value, ethanol content, sulfur, or phenolics in regards to the antimicrobial effect. In fish fillets marinated in wine + water mixture (*v*/*v* = 1:1) and inoculated with 7 log colony forming units (CFU)/25 g the growth of bacteria was reduced after three days of storage at 4 °C. Subsequent storage resulted in the growth of bacteria in all samples, with the lowest growth of *E. coli* ATCC^®^ 25922 in macerated Graševina and *E. coli* ATCC^®^ 8739 in standard Graševina. All wines showed the capacity to reduce the number and growth of heavily infected sea bass filets, but correlation with specific wine constituents was not found. Taking into account the numerous reactive mechanisms between food and wine, all in vitro studies in controlled laboratory conditions should be further verified in the relevant environment, and additional research is needed to clarify the role of individual wine components in the mechanism of antimicrobial activity.

## 1. Introduction

Food-borne bacteria have become an ever-increasing challenge, in both commercial and domestic use. Contaminated food continues to cause millions of people to fall ill, with many succumbing to fatality due to foodborne bacterial infections [1]. *Escherichia coli* are among the most common food-borne pathogens related to food spoilage and food poisoning. *E. coli* are found in the environment and the lower intestines of warm-blooded organisms as a part of normal microflora and represent mostly harmless bacteria. Certain types of *E. coli*, such as shiga-like-toxin-producing *E. coli* (serotype O157:H7, SLTEC), contaminate different types of foods and water, presenting a risk for humans [2,3]. The main sources of human infections are undercooked and contaminated meat and fish, as well as dairy products made from raw milk. Despite various measures taken during processing, consumers can still be exposed to this pathogen by consumption and handling of improperly prepared foods or through cross–contamination as another major risk factor [3,4].

*E. coli* can tolerate and rapidly adapt to a variety of stressful conditions such as fluctuations and extremes of temperature, high osmolarity, and low pH [5]. One potential approach to reducing the counts of *E. coli* could be the application of natural and sustainable substances with antimicrobial properties. Red and white wines have demonstrated antimicrobial activity under various experimental conditions against spoilage and pathogenic food bacteria such as *Bacillus* spp., *Escherichia* spp., *Listeria* spp., *Salmonella* spp., *Sigella*, *Pseudomonas* spp., *Campylobacter jejuni*, *Yersinia enterocolitica*, *Vibrio* spp., and *Staphylococcus aureus* [6,7,8,9,10,11,12,13,14,15,16,17,18,19].

It is generally held that wine’s biological potential is related to a large number of polyphenolic compounds [12]. Polyphenols (phenolics) are plant secondary metabolites, which have essential roles in plant physiology but have potential health benefits on the human organism, mainly as antioxidant, anti-allergic, anti-inflammatory, anticancer, antihypertensive, and antimicrobial agents [20]. The antimicrobial properties of polyphenols have been widely studied to propose new methods of food preservation and enhance food safety without the use of synthetic additives. Among polyphenol classes, *E. coli* showed sensitivity to phenolic acids, hydrolysable and condensed tannins, flavan-3-ols, and flavonols. All of these are found in fruits and vegetables, and plant–derived beverages, such as tea and wine [20]. Red and white wines contain a variety of phenolic compounds that have been associated with numerous health benefits mentioned earlier but have also been active against foodborne pathogens.

In past years, great attention has been paid to wine polyphenols and naturally produced macerated white wines are becoming increasingly popular; however their biological effects are rarely studied. These wines have a high content of biologically active compounds and among them; polyphenols are considered the main factor responsible for particular biological properties. The phenolic composition of wines varies with a grape variety, but it is also influenced by viticultural and environmental factors, and the wine-making process. However, the skins and seeds of grapes are known to be rich sources of polyphenolic compounds, both flavonoids and non-flavonoids [21], and thus phenolic-rich wines are commonly obtained through maceration step, in which grape juice is left in contact with grape skins, seeds and even steams, for weeks and even months [22]. Although wine polyphenols have been intensively studied, it has been demonstrated that the synergistic effect of phenolics with other wine components, such as low pH, ethanol, and organic acids, remains the key mechanism of wine bioactivity [11,12,23]. The specific components of wine and their relative contribution to its antimicrobial activity are still a matter of debate.

There have been a limited number of studies investigating the antimicrobial effect of wine used in the process of food preparation, either as an ingredient or marinade. In available studies, the wine was not tested intact, but in the form of wine extract or in the combination with soya sauce, oregano and thyme essential oils, garlic juice, or even pure phenolic compounds [7,8,9,10,24,25]. Marinade recipes containing red or white wine enriched with plant essential oils demonstrated strong activity under laboratory conditions against major foodborne pathogens [11]. Also, the wine application in foods contributed to the prolongation of shelf life of meat [8] and fish [24] products with the ability to reduce total viable count in the products. Both red and white wines from different regions and grape varieties showed the antimicrobial potential against *E. coli*, however, the in vitro effect was not often confirmed in food models [12,19].

There is an increasing interest in investigations into the use of bioactive natural compounds for the enhancement of food safety and product quality. Microbiological challenge tests are an important tool necessary for simulation of assessment of real-life food safety and prediction of safety risks. The use of natural antimicrobials and preservative sources in foods such as wine would help reduce the use of synthetic additives and aggressive processing, without compromising food safety. In this regard, the main objective of the present study was to test the antimicrobial activity of five wines against two *E. coli* strains and to verify the findings in wine-marinated fish fillets as a food matrix.

## 2. Materials and Methods

### 2.1. Wine Samples and Technology

**Figure 1:**
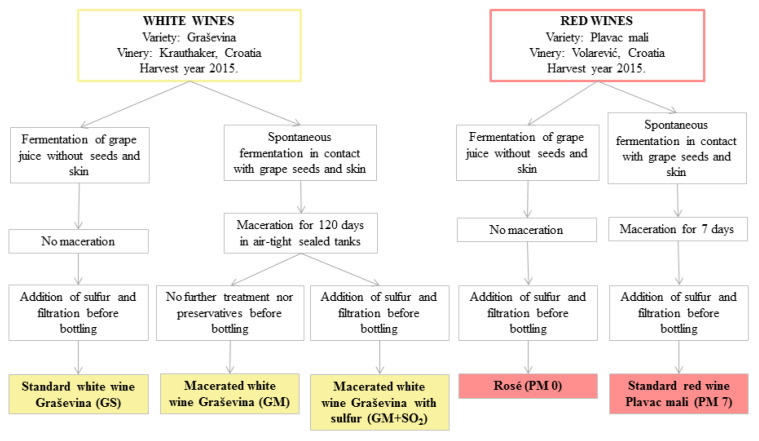
Technology of the tested wines.

The five wines, three white wines produced from the grape variety Graševina in 2015 at the Krauthaker winery, and one red and one rosé produced from the grape variety Plavac Mali in 2015 at the Volarević winery, Croatia, were tested in vitro for antimicrobial activity in the standard growing media and in fish fillets as food matrix. The technology of production of the wines tested is schematically shown in Figure 1. The standard white wine (GS) and rosé wine (PM 0) were produced following a classic methodology requiring grape juice to be separated from the hard parts of the grapes during fermentation, without maceration. Conversely, the Georgian wine production principles were used in obtaining macerated white wines rich in polyphenols. Following spontaneous fermentation, and without removing grape seeds and skins, the tanks were sealed air-tight, allowing the wine further elaboration for four months. Part of the wine so produced was bottled without further treatment (GM), while sulphur (GM + SO_2_) was added to the other part as a preservative. The red wine (PM 7) production process included both fermentation and maceration for seven days in contact with the grape seeds and skins (Figure 1).

### 2.2. Wine Chemistry Analyses

The enological analysis of all wines included ethanol and sulfur content, along with pH and acidity values. These were determined according to the Compendium of International Methods of Analysis of Wines and Musts of the International Organisation of Vine and Wine (OIV), and are shown in Table 1.

### 2.3. Analysis of Total Phenolics, Tannins and Anthocyanins

The total phenolic content of the wine samples was measured spectrophotometrically using the Folin-Ciocalteu method [21,26] with the results expressed as mg of gallic acid equivalents per liter (mg GAE/L).

The total tannin contents were measured by acidic hydrolysis of proanthocyanidins resulting in carbocation formation with partial conversion into red cyanidin using the method of Ribéreau-Gayon and Stonestreet [27] and concentration was obtained in g/L.

Anthocyanin content was determined by the SO_2_ bleaching procedure and results are expressed as mg/L [28].

All measurements were carried out in triplicate using a SPECORD 200 Plus (Analytik Jena AG, Jena, Germany).

### 2.4. The Escherichia coli Strains and Inoculum Preparation

Bacterial strains *E. coli* WDCM00012 (ATCC8739, *E. coli* 12) and WDCM00013 (ATCC25922, *E. coli* 13) were used as test microorganisms. Determination of antimicrobial activity was performed using Müeller Hinton Broth/Agar (MHB, MHA; Biolife, Italy). The cell culture suspension was prepared by selection of a colony from the 24-hr old MHA plates, with adjustment in MHB to include a concentration of approximately 10^8^ colony forming units per mL (CFU/mL) according to the turbidity of 0.5 McFarland scale standard (Densomat, bioMerieux, Marcy l’Etoile, France). An aliquot of 2 mL of cell culture suspension was diluted with 38 mL of MHB medium for inoculum preparation (initial concentration 10^7^ CFU/mL).

### 2.5. In Vitro Evaluation of Antimicrobial Activity of Wines

#### 2.5.1. Agar-Disk and Agar-Well Diffusion Assays

The protocols used for agar-disk and agar-well diffusion assays are defined by Clinical and Laboratory Standards Institute (CLSI) [29,30]. The Petri plates containing 20 mL MHA were inoculated by spreading microbial inoculum over the entire agar surface using sterile cotton swabs rolled in the inoculum. For the disk diffusion method, filter paper discs of 6 mm in diameter containing 25 µL of the wine samples were placed on the agar surface. The same was done for the antibiotics, chloramphenicol, gentamicin, and tigecycline, used as control. The Petri plates were incubated at 37 °C for 20–24 h. The inhibition zone of the antibiotics was in the range proposed by CLSI and the European Committee on Antimicrobial Susceptibility Testing (EUCAST) [30,31] for both *E. coli* strains.

For agar-well diffusion assays, four equidistant holes were made in the agar using sterile cork borers (Ø = 7 mm) and 50 µL of the wine samples was added directly in the wells. The agar plates were at 4 °C for one hour, following incubation at 37 °C for 20–24 h. The inhibition zones formed on the medium were measured in millimeters (mm) [32]. If the inhibition zone was ≥12 mm, the tested sample was considered to have good inhibitory effect [33]. All measurements were done in triplicate with two positive growth controls included (ethanol in the concentration of 13–15%).

#### 2.5.2. Minimal Inhibitory Concentration (MIC)

Minimal inhibitory concentration was measured according to the previously described method by [32,34]. Shortly 50 µL of bacterial inoculum and 50 µL of two-fold serially diluted wine samples were added to the wells of a sterile 96-well microtitare plate. Three antibiotics (chloramphenicol, gentamicin, and tigecycline) and ethanol (13–15% vol) were used as control. The MIC values of chloramphenicol, gentamicin, and tigecycline were in the range proposed by CLSI and EUCAST standards, 4, 0.5 and 0.15 µg/mL, respectively [30,31]. The MIC was defined as the lowest concentration displaying the ability to inhibit any visible bacterial growth after adding 10 µL/well of INT (2-p-iodophenyl-3-p-nitrophenyl-5-phenyl tetrazolium chloride, Sigma, Darmstadt, Germany). All measurements of MIC values were done in triplicate and expressed as mg GAE/L.

### 2.6. Preparation of Fish Samples and Inoculation with E. coli

Seabass (*Dicentrarchus labrax*) were purchased at the local fish market and transported to the lab packed with ice in polystyrene boxes. The fish were of class L, weighing ~400–600 g each. All further preparations were performed under sterile conditions. The fish was filleted and the fillets thoroughly washed in sterile distilled water. Five groups of fish fillets, each weighing approximately 200 g, were marinated in a mixture of wine and sterile distilled water (400 mL) prepared in the ratio 1:1. The control sample was kept in sterile distilled water. After two hours, each group of fillets was drained and ground in a knife mill down to 40–50 mm particles (GRINDOMIX GM 200, Retsch GmbH, Haan, Germany). Next, 50 g of ground meat was separated for pH measurement.

For the challenge test experiment, triplicate packages containing chunks of 25 ± 0.01 g of ground wine-marinated fish meat were placed in sterile vacuum bags, inoculated with *E. coli* strains (0.5 mL of liquid inoculum prepared as described in Section 2.4) to achieve an initial value of ~7 log CFU/ 25 g and vacuum sealed. The samples were kept at room temperature for 10 min to allow possible attachment and diffusion [7] and then stored at 4 ± 1 °C. The number of *E. coli* was enumerated after three and seven days. In addition, triplicate packages of wine-marinated fish meat without inoculum were teste for *E. coli*.

#### 2.6.1. Enumeration of *E. coli*

The enumeration of *E. coli* was done using ISO methodology [35]. Briefly, the samples were transferred to stomacher bags with 225 mL of sterile peptone water and homogenized using a laboratory blender (Stomacher, Maxicator, IUL S.A., Barcelona, Spain) for three minutes. The serial of decimal dilutions was prepared and distributed to sterile Petri dish followed by the Tryptone Bile X-GLUC agar (Biolife, Milan, Italy) and incubated at 44 °C for 24–48 h. Plates with 10 to 150 blue-green colonies were counted. Confirmation of *E. coli* was done by Colilert-18 (Quanti-Tray/2000) method. According to the manufacturer’s instructions, yellow wells indicated coliform bacteria, and wells that were yellow and fluorescent when exposed to UV light (366 nm) indicated *E. coli*. Results were expressed as log CFU/25 g based upon the average from triplicate plates.

#### 2.6.2. pH Analyses

The pH was measured in a mixture of non-inoculated ground meat and distilled water (ratio 1:10) using a digital pH meter (Easy Five 720, Mettler Toledo, Switzerland) calibrated at three points (4, 7 and 10) [36]. Measurements were performed in triplicate.

### 2.7. Statistical Analyses

The statistical differences between chemical parameters were determined by analysis of variance (one-way ANOVA) followed by a least significant difference test at a 95% confidence level.

## 3. Results and Discussion

### 3.1. Wine Chemistry

The results of chemical analyses of the wines are shown in Table 1. The chemical parameters, alcohol, pH, and acidity in the wines were in the range of 12.8–14.3 vol%, 3.5–3.9, and 3.8–5.7 g/L respectively. Except for GM, the SO_2_ was within the allowed limits (150 mg/L for red wines, 200 mg/L for white and rosé wines) [37]. Being a very complex process, winemaking involves steps that enrich the wine with components that enhance the flavor, color, and aroma. One such step, the maceration, has the purpose of extracting such compounds from the hard parts of grapes, which are left in contact with the grape juice during fermentation and later during the elaboration of the wine. For example, seven days of maceration resulted in a much higher anthocyanin content in red wine relative to the rosé obtained from the same grape. Similarly, the concentration of total phenols (TP) and tannins significantly increased with maceration, both in white and red wine (Table 1). The phenolic composition of the wine undergoes significant changes during the crushing of grapes, maceration, and fermentation [12,22,25].

In macerated Graševina and Plavac Mali, the concentration of total phenolics was previously reported to be 2699–2850 mg GAE/L and 3210 mg GAE/L, respectively, which is significantly higher in comparison to standard Graševina (305 mg GAE/L) and Plavac Mali Rosé (1074 mg GAE/L) [22].

Phenolic compounds participate in developing the sensory characteristics of wine, such as color, astringency, and bitterness, with most of these compounds having a wide range of biological properties including antimicrobial [9,10,11,20,25]. Some reports indicate that phenolics from wines are probably the most active components in inhibiting the growth of pathogens [10]. On the other hand, the synergy of different compounds such as ethanol, phenolics, certain organic acids, SO_2_, and low pH in reducing the bacterial count seems to remain the key to wine bioactivity [6,7,11,13].

### 3.2. Antimicrobial Activity

The results of the antimicrobial activity of wines are shown in Table 2. The MIC values show the minimal concentration of wine needed to kill *E. coli*, while the results of the agar-well diffusion method show the ability of the wine to inhibit the growth of inoculated bacteria. The wines demonstrated antimicrobial effect against both *E. coli* strains. The two wines with the lowest phenolic content (GS and PM 0) had higher antimicrobial activity in comparison with the other wines. Aside from the small concentration of total phenolics (Table 1), these two wines had the highest content in total (124–173 mg/L) and free SO_2_ (9–27 mg/L) previously pointed out as a possible inhibitor of bacterial growth in the grape juice and wine [13]. As the only wine with no added SO_2_, GM showed the lowest antimicrobial activity. Although studies showed a contribution of malic and tartaric acid in the inactivation of pathogens, particularly with the low surrounding pH (≤3) [6,13], our results demonstrated that the wines with the same acidity (GS and GM) have significantly different MIC values (Table 2). On the other hand, PM 0 with the lowest acidity value (Table 1) showed good antimicrobial activity. It is important to note that phenolics alone are certainly not responsible for the antimicrobial effect of the wines. PM7 is the only wine that showed different MIC values for the two strains, with *E. coli* 12 being more sensitive; however, the mechanism behind this result was not determined. It can only be assumed that a particular group of compounds present in PM7 has more than one mechanism of action (discussed later in the text) on the bacterium resulting in a better antimicrobial effect.

Using the agar-disk and agar-well diffusion methods, we could not demonstrate antimicrobial effectiveness for any of the tested wines. Inhibition zones were not detected around the discs and around the wells they were <8 mm, which indicates no inhibitory effect [33]. The small volume of wine (25 and 50 µL) and a correspondingly small amount of potentially antimicrobial wine components might be why the effect was not recorded. This brings to question the suitability of these methods for testing the antimicrobial activity of the wines. On the other hand, MIC values showed accurate, reproducible, and reliable results.

We could not establish the specific relationship between the winemaking process and a particular MIC value, taking into account the pH value, ethanol content, SO_2_, or total phenolics. The findings rather indicate the importance of their synergy for the observed overall antimicrobial effect of the tested wines. The maceration duration and the addition of sulfur in the vinification process could not be fully correlated with the antimicrobial effects of the wines. This was also previously confirmed in several studies [6,12,14]. Further, the ethanol diluted to the level of concentration found in the wines (13 to 15%) showed no antimicrobial activity. Previous in vitro studies demonstrated that ethanol concentration in the wine (usually between 10 and 13% *v*/*v*) is considerably low to account for bactericidal effect [6,13,38]. The bactericidal impact of the wine itself, for a given ethanol concentration, is more effective than any other alcoholic beverage. Some authors find that the combination of organic acids (lactic, malic, acidic, and tartaric) and ethanol contribute to this stronger antimicrobial effect of the wine [38,39,40].

The particular role of total phenolics remains unclear. In previous studies, it has been suggested that compounds such as flavonoids (quercetin and quercetin-3-glucoside) and monomeric anthocyanins might be used as biochemical markers that contribute to the antimicrobial activity of red wines [9,14]. Among wine phenolics, resveratrol has been particularly emphasized as a strong antimicrobial compound [34,41]. In line with our results, Boban et al. [42] showed that resveratrol levels in the intact, dealcoholized, and thermally treated wines at 125 °C, which were similar in pH, total phenolics and ethanol content, differed significantly, although the wines showed a similar antimicrobial effect. To clarify the role of polyphenols, pH, ethanol, and other wine components, a study tested the antimicrobial effects of intact wine in comparison to that of phenols-stripped wine, dealcoholized wine, ethanol, and low pH applied separately and in combination [23]. Antibacterial activity of the samples could not be related to their total phenolics and resveratrol content, ethanol content, or pH. After intact wine, the phenols-stripped wine had the strongest antimicrobial effect against *S. enterica* and *E. coli*, so the authors concluded that nonphenolic constituents of wine were responsible for a major part of its antimicrobial activity. Further studies are needed to clarify the mechanism of antimicrobial action of phenolic compounds and their correlation with other chemical parameters, but it can be concluded that the antimicrobial activity of a complex solution such as wine is based on more than one compound.

### 3.3. The Experiment on Fish Samples

The pH values of the wine-marinated fish meat used for the inoculation of bacteria are shown in Table 3.

In wine-marinated fish meat without inoculum, the number of CFU/25 g was zero in all samples. The total viable bacterial count or other bacteria genera were not analyzed in raw material or during the experiment.

**Figure 2:**
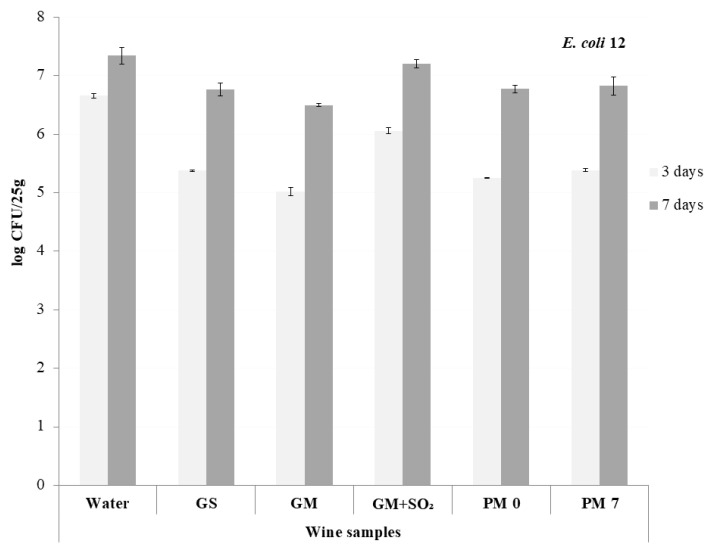
Count of *E. coli* (WDCM00012) in wine-marinated seabass (*Dicentrarchus labrax*); (GS—standard white wine, GM and GM + SO_2_—macetared white wine with and without SO_2_, PM 0—rosé, PM 7—macerated redwine) after 3 and 7 days of storage at 4 ± 1 °C.

**Figure 3:**
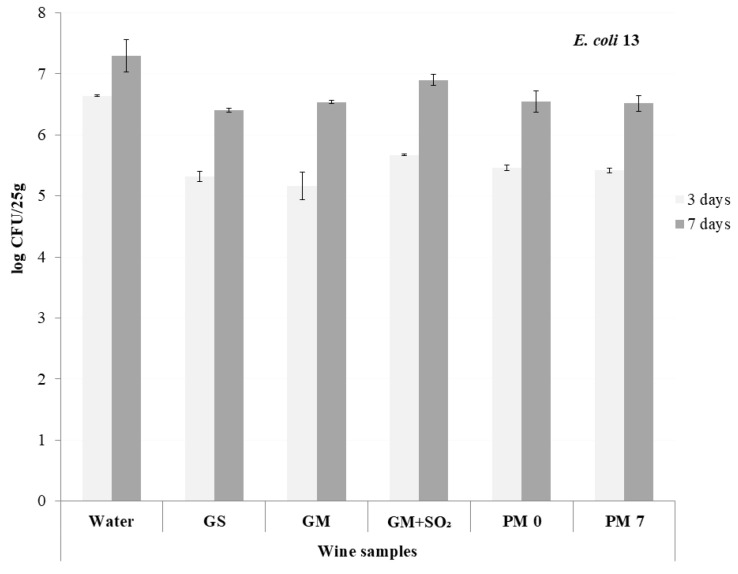
Count of *E. coli* (WDCM00013) in wine-marinated seabass (*Dicentrarchus labrax*); (GS—standard white wine, GM and GM + SO_2_—macetared white wine with and without SO_2_, PM 0—rosé, PM 7—macerated redwine) after 3 and 7 days of storage at 4 ± 1 °C.

Three days after the inoculation of the wine-marinated fish fillets with *E coli* strains, which were held at 4 ± 1 °C, the initial loads were <7 log CFU/25 g in all samples (Figure 2 and Figure 3). After three days of storage, the levels of both *E. coli* strains were the lowest in GM (biggest MIC values and no added sulfur in winemaking process) and GS samples, followed by the rosé and standard red wines (PM 0 and PM 7).

The chemical parameters of wine samples could not be correlated with their antimicrobial activity. The 200 mL of wine that was used for marinating the fillets contained five times less TP than reported in Table 1, accordingly 61, 540, 570, 215, and 642 mg GAE in GS, GM, GM + SO_2_, PM 0, and PM 7, respectively. For GS, GM, and PM 0 the MIC values obtained are presented below, however, these wines showed better inhibition against *E. coli* strains than GM + SO_2_ which contained the inhibitory concentration of TP in the 200 mL and high levels of SO_2_. PM 7, with the highest TP content (including anthocyanins and tannins), showed similar inhibition strength as other wines, with no difference between the two strains regardless of the MIC values. The pH with the contribution of ethanol was found to be an important factor in predicting in vitro inactivation and the efficacy of wine treatments against *E. coli* [6]. Our results show that this contribution is not valid for food model testing.

Subsequent storage resulted in the growth of bacteria in all samples, from 0.6 to 1.5 log cycles in samples inoculated with *E. coli* 12, and from 0.6 to 1.4 log cycles in *E. coli* 13. After seven days of storage, the number of *E. coli* 12 was the lowest in GM and *E. coli* 13 in GS. The effect of rosé and red wines (PM 0 and PM 7) was similar, and the growth was again the highest in GM + SO_2_. In comparison to the control, all wines showed the capacity to reduce the number and growth of heavily infected sea bass filets. The role of SO_2_ in the antimicrobial activity of wine is questionable; its purpose in wine seems to be mainly antioxidant (prevention of browning) [22].

The specific spoilage bacteria such as Pseudomonas and H_2_S-producing are mostly predominant in the spoilage flora of chilled seabass, causing off-flavors, and sensory rejection. Generally, the number of *Enterobacteriaceae* counts in chilled seabass is around 2 log CFU/g, and remain at low levels until ca. 7–9 days of storage [43]. The antimicrobial effect of phenolics against these microorganisms is usually concentration-dependent [44]. It has been reported that the phenolics in wine inhibit the growth of bacteria by multiple mechanisms, including membrane damage, inhibition of nucleic acid synthesis, inhibition of metabolism, and inhibition of cell wall and cell membrane synthesis [45]. The catechin was reported to retard the growth of spoilage bacteria in fish products [44], also high-tannin extracts were found to inhibit growth, in contrast to high-flavonoid extracts, which were found to induce bacterial growth [19]. There have been few studies conducted pertaining to bacterial survival, especially *E. coli* in food models with wine. Boban et al. [42] found that the intact red wine kills *S. enterica* and *E. coli* after direct exposure in less than 5 min. In addition, the direct exposure of bacterial cells to white and red wine resulted in the rapid inactivation of *C. jejuni* [7]. The white and red wine marinades have shown beneficial effects against meat products contaminated with different foodborne pathogens [7,15,16,18]. In all of the studies mentioned, the wine was used as one of the ingredients of the marinades, and its antimicrobial effect was tested in combination with salt, spices, plant extracts, and essential oils. To our knowledge, there is no available literature on the challenge tests against *E. coli* using wine as the main antimicrobial substance. Being a source of the numerous bioactive compounds, it is hard to separate one distinctive segment responsible for wine’s antimicrobial activity. The results showed sufficient evidence that the wines should be further investigated to relate the antimicrobial properties with its various compounds [38].

## 4. Conclusions

This research aimed to evaluate the antimicrobial effect of different wines, produced using different technologies, against two strains of *E. coli*, and the strains’ behavior in wine marinated seabass fillets. The study gives evidence that all wines have antimicrobial effects in vitro and in food models, and indicate that exposure to wine may inhibit the growth of the strains; however, the extrapolation of the results when the foods are contaminated with *E. coli* should be taken with reserve since a low number of strains were tested, invasiveness of the strains after treatment with wine was not determined, and the inhibitory effect was obtained during a short period of storage. Taking into account that in a complex nutrient medium such as fish, there are numerous reactive mechanisms between food and wine; thus the food matrix may have some buffering capacity which can reduce the antimicrobial effect. Further studies should involve other bacterial strains and genera, investigations of different microbial virulence factors, and antimicrobial effects of individual phenolic compounds and their mixtures to uncover mechanisms involved in the antimicrobial response.

## Figures and Tables

**Table 1:** Chemical composition of tested wines.

Wine Samples *	pH	Acidity (g/L) **	Alcohol (%)	Total Phenolics (mg GAE/L)	Anthocyanins (mg/L)	Tannins (mg/L)	Free SO_2_ (mg/L)	Total SO_2_ (mg/L)
GS	3.54	4.9	13.0	305 ± 3.5 ^a^	0 ^a^	0.26 ± 0.01 ^a^	27	124
GM	3.94	4.8	13.3	2699 ± 8.2 ^b^	0 ^b^	3.34 ± 0.10 ^b^	1	3
GM + SO_2_	3.90	5.4	12.8	2850 ± 34.6 ^b^	0 ^c^	4.93 ± 0.19 ^c^	5	99
PM 0	3.54	3.8	14.3	539 ± 22.2 ^c^	1.97 ± 0.04 ^b^	1.08 ± 0.07 ^d^	9	173
PM 7	3.61	5.7	13.7	1988.96 ± 35.0 ^d^	30.14 ± 6.52 ^d^	4.93 ± 0.46 ^e^	6	89

* GS—standard white wine, GM and GM + SO_2_—macerated white wine with and without SO_2_, PM 0—rosé, PM 7—macerated red wine: ** expressed as tartaric acid; ^a–e^ mean value ± standard deviation in the same column followed by different superscript are significantly different (*p* < 0.05).

**Table 2:** Antimicrobial activity of wines (*n* = 6).

	MIC (mg GAE/L)		Well (mm)	
Wine Samples *	*E. coli* 12	*E. coli* 13	*E. coli* 12	*E. coli* 13
GS	122.0	122.0	<8	<8
GM	1079.6	1079.6	<8	<8
GM + SO_2_	570.0	570.0	<8	<8
PM 0	215.6	215.6	<8	<8
PM 7	397.8	795.6	<8	<8
Ethanol (13–15%)	0	0	<8	<8

* GS—standard white wine, GM and GM + SO_2_—macetared white wine with and without SO_2_, PM 0—rosé, PM 7—macerated redwine.

**Table 3:** The pH of the wine-marinated fish meat (*n* = 3).

Wine Samples *	pH
Water	6.58 ± 0.01 ^a^
GS	6.27 ± 0.02 ^b^
GM	6.32 ± 0.01 ^b^
GM + SO_2_	6.40 ± 0.01 ^c^
PM 0	6.25 ± 0.01 ^b^
PM 7	6.28 ± 0.01 ^b^

* GS—standard white wine, GM and GM + SO_2_—macerated white wine with and without SO_2_, PM 0—rosé, PM 7—macerated red wine; ^a–c^ means followed by different superscript are significantly different (*p* < 0.05).
